# (In)action on the social and commercial determinants of health: a call to arms to push the agenda forward

**DOI:** 10.1177/17579139241277434

**Published:** 2025-09-23

**Authors:** RJ Noonan

**Affiliations:** Faculty of Health and Wellbeing, University of Bolton, Deane Road, Bolton BL3 5AB, UK

## Abstract

In this article, Noonan contends that boosting public support is vital for increasing pressure on the Government to enact change for the common good. He argues that the public health community can build public support for the required structural changes by challenging misinformation and sharing a new story.

Health is a precious asset. Health also functions as a social barometer. Disease rates, life expectancy, and years lived in good health offer indication of how well a society is functioning. In the United Kingdom (UK), there are clear signs that society could be functioning better. Life expectancy is stalling and, in some cases, falling.^1^ Rates of obesity, psychological distress, and deaths of despair are rising. Health inequalities are widening, and more and more people are treading water every day, trying to survive.^2,3^

Where we live, the job we do and the money we earn influence the opportunities we have available to us to be healthy. These factors have become known as the social determinants of health. In his landmark report, *Fair Society, Healthy Lives*,^4^ Sir Michael Marmot proposed an evidence-based plan to tackle health inequalities. Key to his plan was action on the social determinants of health. Essentially, Marmot’s report, like many of the others before it,^5,6^ contended that the solution to poor health is to prevent it from occurring in the first place. It involves replacing the short-term, plaster sticking approaches with long-term preventative approaches to tackle the root cause of poor health. The focus has to be on increasing people’s opportunities to live well; creating more favourable living and working conditions that enable people to take control over their lives.

Politicians often say that their decisions will be evidence-led. However, in the case of health, this is not entirely true. That political discourse continues to frame health solely in the context of individual choices which overlooks overwhelming evidence on the social determinants of health, including Marmot’s work.^2,4^ It also overlooks evidence on the commercial determinants of health (i.e. strategies used by industry to promote products that are harmful to health).^7^ The only solution put forward by politicians to improve people’s health is for there to be better education around healthy lifestyles. The fact that they usher such a narrative only goes to reinforce the widespread public perception that ‘staying healthy’ is an individual responsibility.^8^

Industry is a key obstacle to truth about the things that matter most to our health. It is also an obstacle to action on the wider structural changes needed to enable people to live healthily. Industry impacts population health in a myriad of ways.^9^ This reality was captured in the World Health Organisation’s (WHO) most recent report in which they blamed the tobacco, alcohol, processed food, and fossil fuel industries for almost 3 million deaths every year in Europe.^10^ Not only do these industries advertise and sell products that harm health,^11^ they quash evidence and lobby against government policies and public health interventions aimed at protecting the public’s health. It took decades for evidence linking tobacco and non-communicable diseases to be accepted in public and political discourse due to evidence quashing and relentless lobbying from the tobacco industry.^12^ The WHO report offers striking evidence on how the industry continues to use these same tactics to undermine public health policies. The industries with the most to lose from tougher regulations have the greatest incentive to invest in lobbying; and they do invest. Every year they spend billions on lobbying in the UK alone.

The Government has a responsibility to help people stay healthy and protect them from harm owing to power imbalances. They are failing to fulfil this responsibility because they are fixated on growing the economy. As a nation, we continue to value wealth over health rather than health over wealth.^13^ There are various immediate steps the Government are able to take to better protect people from the harms of *free market* failure. They can stand up to industry by implementing tougher regulation and taxation on harmful products. They can also invest more in health and public services, and they can adopt fairer, more progressive tax systems to better redistribute income and wealth.

Public support is vital for increasing pressure on government to enact change for the common good. One underexplored path to progress in this respect is through promoting self-awareness and providing the public with an alternative narrative to the status quo. In the words of John McMurtry, ‘Knowledge always win in the end, but not unless and until it is known’.^14^

Over a century has passed since the publication of *The Ragged Trousered Philanthropists*, in which the character, Frank Owen struggled to convince his fellow workers that an alternative future is not only possible but also essential to tackling the *real* causes of their suffering (i.e. poverty).^15^ At a time when the nation is getting sicker and in-work poverty is rising,^3,13^ there remains widespread acceptance across society that ‘*there is no alternative*’ to economic liberalism.^16,17^ However, alternative paths are always possible once imagination and political will are realised. The United States’ war mobilisation in the 1940s is a case in point; not only did it lead to drastic changes in income distribution but it also directed patterns of production and consumption based on the nation’s priorities. Furthermore, it was only four decades ago that the then-UK Government set about *changing the heart and soul* of the British people through economic policy; lowering taxes, cutting regulation and slashing public spending.

Communicating evidence and facts alone will not be enough to build public support for the structural changes needed to enable people to live healthily. We will need an accompanying story, a story that frames health as an asset and exposes the truth that what surrounds us shapes us, including our health. Public health professionals are well placed to promote this new story because people are more likely to act on information delivered by experts.

As a public health community, we have a responsibility to confront and communicate uncomfortable truths. The sharing of alternative discourses will give people hope that a healthier, more socially just and more sustainable future is not just possible, but essential. To protect our collective future, we must challenge misinformation and make hope possible. Change is possible, but only if we restore unity and stand together.
